# Whole-Brain Radiotherapy vs. Localized Radiotherapy after Resection of Brain Metastases in the Era of Targeted Therapy: A Retrospective Study

**DOI:** 10.3390/cancers13184711

**Published:** 2021-09-20

**Authors:** Jaho Koo, Tae Hoon Roh, Sang Ryul Lee, Jaesung Heo, Young-Taek Oh, Se-Hyuk Kim

**Affiliations:** 1 Gamma Knife Center, Brain Tumor Center, Department of Neurosurgery, Ajou University Hospital, Ajou University School of Medicine, Suwon 16499, Korea; jaho9188@gmail.com (J.K.); sangryul79@gmail.com (S.R.L.); nsksh@ajou.ac.kr (S.-H.K.); 2 Brain Tumor Center, Department of Radiation Oncology, Ajou University Hospital, Ajou University School of Medicine, Suwon 16499, Korea; mobilehealth@aumc.ac.kr (J.H.); ohyoung@ajou.ac.kr (Y.-T.O.)

**Keywords:** brain metastases, radiosurgery, targeted therapy, survival, surgery

## Abstract

**Simple Summary:**

The paradigm shift from cytotoxic chemotherapy to molecular targeted therapy dramatically improved the survival and quality of life of cancer patients. In radio-oncological aspects, there also was a paradigm shift from whole-brain radiotherapy to localized radiotherapy including stereotactic radiosurgery. This retrospective study analyzed 124 consecutive patients who had undergone surgical resection of brain metastases. We found targeted therapies to improve overall survival and distant control with decreased incidence of leptomeningeal metastasis. Our data suggest that localized radiotherapy is sufficient after resection of brain metastases when systemic targeted therapy is available.

**Abstract:**

Whether targeted therapy (TT) and radiotherapy impact survival after resection of brain metastases (BM) is unknown. The purpose of this study was to analyze the factors affecting overall survival (OS), local control (LC), distant control (DC), and leptomeningeal metastases (LMM) in patients who had undergone resection of BM. We retrospectively analyzed 124 consecutive patients who had undergone resection of BM between 2004 and 2020. Patient information about age, sex, Karnofsky Performance Scale (KPS), origin of cancer, synchronicity, tumor size, status of primary cancer, use of TT, extent of resection, and postoperative radiotherapy was collected. Radiation therapy was categorized into whole-brain radiotherapy (WBRT), localized radiotherapy (local brain radiotherapy or stereotactic radiosurgery (LBRT/SRS)), and no radiation. We identified factors that affect OS, LC, DC, and LMM. In multivariable analysis, significant factors for OS were higher KPS score (≥90) (HR 0.53, *p* = 0.011), use of TT (HR 0.43, *p* = 0.001), controlled primary disease (HR 0.63, *p* = 0.047), and single BM (HR 0.55, *p* = 0.016). Significant factors for LC were gross total resection (HR 0.29, *p* = 0.014) and origin of cancer (*p* = 0.041). Both WBRT and LBRT/SRS showed superior LC than no radiation (HR 0.32, *p* = 0.034 and HR 0.38, *p* = 0.018, respectively). Significant factors for DC were use of TT (HR 0.54, *p* = 0.022) and single BM (HR 0.47, *p* = 0.004). Reduced incidence of LMM was associated with use of TT (HR 0.42, *p* = 0.038), synchronicity (HR 0.25, *p* = 0.028), and controlled primary cancer (HR 0.44, *p* = 0.047). TT was associated with prolonged OS, improved DC, and reduced LMM in resected BM patients. WBRT and LBRT/SRS showed similar benefits on LC. Considering the extended survival of cancer patients and the long-term effect of WBRT on cognitive function, LBRT/SRS appears to be a good option after resection of BM.

## 1. Introduction

Brain metastasis (BM) occurs in 20–40% of cancer patients and results in shorter survival and poor quality of life [1,2]. The incidence of BM is increasing, with prolonged survival owing to the development of cancer therapies such as targeted therapy (TT) [3].

For small BMs without symptoms, radiotherapy or radiosurgery is a mainstay for treatment. Whole-brain radiotherapy (WBRT) was the first standard therapy for BM and showed survival benefits [4]. Stereotactic radiosurgery (SRS) also proved to have survival benefits in patients with 1–10 BMs [5]. WBRT followed SRS to prevent distant metastases, but many studies have questioned the role of additional WBRT after SRS because it shows no clear survival benefit [6]. Moreover, WBRT was related to cognitive impairment and poor quality of life [7].

Surgical resection is a standard treatment for a single BM, especially when it is large in size and causes a mass effect or obstructive hydrocephalus [8,9,10]. However, patients who had undergone surgical treatment for BM were exposed to a higher risk of leptomeningeal metastases (LMM) compared with patients who had received SRS [11,12,13]. WBRT can prevent local and distant recurrences, LMM, and death due to neurological causes in such patients [4,11]. However, since the death of cancer patients was dependent on systemic progression of disease, WBRT after resection of BM did not show a clear survival benefit [6].

Cognitive decline and neurotoxicity after WBRT have shifted attention to localized radiotherapy, such as SRS, as an alternative treatment [14]. Furthermore, fractionated stereotactic radiation therapy (FSRT) has emerged as a treatment for large BM [15]. Moreover, local brain radiotherapy (LBRT) to the resection cavity showed similar LC compared with WBRT [16,17,18].

Recently, advances in molecular TT and immune checkpoint inhibitors dramatically improved the survival of some cancer patients. Thus, the quality of life of cancer patients is of more concern than in the past. Many centers are deferring WBRT after resection of BM, while adopting localized radiotherapy such as LBRT, FSRT, or SRS [19]. However, little is known about the differences in long-term outcomes of these modalities. Furthermore, the effects of TT on postoperative recurrences are not well known. In this study, we analyzed and identified the factors affecting overall survival (OS), local control (LC), distal control (DC), and leptomeningeal metastasis (LMM) in patients who had undergone surgical resection of BM.

## 2. Materials and Methods

We retrospectively analyzed consecutive patients who had undergone surgical treatment for metastatic brain tumors in our institute between June 2004 and December 2020. Patient information on age, sex, Karnofsky Performance Scale (KPS) score, day of operation, method of radiation therapy (fraction, dose, time), and status of primary cancer was collected from electronic medical records. Information on extent of resection and number of BMs also was collected. Indications for surgical resection for BM were the presence of related neurological symptoms or mass effects in a surgically accessible location. If there were multiple BMs, only symptomatic lesions were resected. KPS scores were measured based on preoperative conditions. Postoperative treatment modality was determined through a multidisciplinary conference in each case. The presence of recurrence was determined based on RANO-BM criteria [20]. LMM was determined by brain or spine magnetic resonance image (MRI) or cerebrospinal fluid cytology. Brain MRI was performed every 3 months after surgery in patients with residual lesions and every 6 months if the patient had no symptoms or was stable. The OS was defined as the time from surgery to last visit or death. Follow-up duration and recurrence were based on the last MRI performed from the date of surgery. Distant recurrence was defined as a case of a new lesion distant from the resected cavity.

Radiation therapy was divided into three groups of LBRT/SRS, WBRT, and no radiation. The LBRT/SRS group comprised patients who had received radiation other than WBRT. Although fractionation of radiation varied depending on size of the resection cavity, a similar biologically equivalent dose (BED) was applied across the LBRT/SRS group. We grouped these localized radiotherapies as a counterpart to WBRT. For LBRT, patients underwent a simulation in the supine position with a mask device using a computed tomography (CT) scanner with 3 mm slice intervals for three-dimensional RT planning. The initial clinical target volume (CTV) contained an entire resection cavity. The planning target volume (PTV) was defined as the CTV plus an at least 2 mm margin. This margin was modified around natural barriers such as the skull, ventricles, and falx. Organs at risk (OAR) were defined as the brain stem, optic nerves, and optic chiasm. For SRS, patients were treated with the Leksell GammaKnife^®^ (Model C or Icon™; Elekta AB, Stockholm, Sweden) either fixed by Leksell Frame for single session or by thermoplastic mask for multiple sessions. The prescription dose was determined by diameter of the resection cavity. Resection cavities with diameters <1, 1–2, 2–3, and >3 cm received 20, 18–20, 16–18, and 12–16 Gy, respectively.

In contrast with cytotoxic chemotherapy, patients treated postoperatively with any drugs that target specific molecular pathways including cancer-driving mutation, growth factors, transcription factors, hormone receptors, or immune checkpoint were grouped as TT in our study.

The survival was calculated using the Kaplan–Meier estimate and compared between groups using a log-rank test. A Cox proportional hazards model was used for univariable and multivariable analyses for OS, LC, DC, and LMM.

All research was performed following institutional guidelines and the Declaration of Helsinki of 1975 in its most recent version. The institutional review board permitted this study. Patient consent was waived due to the retrospective nature of this study.

## 3. Results

### 3.1. Patient and Treatment Characteristics

One hundred fifty-nine patients received surgery for BM between 2004 and 2020. A total of 35 patients were excluded because 19 died within three months, and 16 were lost to follow-up. One hundred twenty-four patients were included in the analysis. A total of 26 (21.0%) patients received LBRT, and 37 (29.8%) received single-session SRS to the resection cavity. Thus, 63 (50.8%) patients were included in the LBRT/SRS group. A total of 24 (19.4%) patients received WBRT, and 37 (29.8%) did not receive adjuvant radiation therapy after surgery for BM. No patient received localized radiotherapy plus WBRT before recurrence. The median number of fractions of LBRT was 10 (range 3–29), and the median radiation dose was 30 Gy. The median imaging follow-up period for patients without local recurrence was 8.2 months (range: 0.4–105.4 months). The median treatment time from surgery to adjuvant radiation therapy was 15 days (range: 0–63 days) for LBRT/SRS and 19 days (range: 0–28 days) for WBRT.

Patient demographics are indicated in Table 1. The median age was 57.7 years (range: 28–78 years), and the median KPS score was 90 (range: 30–100). Primary disease was located in lung (50, 40.3%), breast (34, 27.4%), gastrointestinal tract (12, 9.7%), ovarian, uterus or cervix (10, 8.1%), genitourinary tract (6, 4.8%), head and neck (5, 4.0%), hepatobiliary (4, 3.2%), and melanoma (3, 2.4%). Of the patients, 91 had metachronous BM (73.4%) and 33 had synchronous BM (26.6%). The mean preoperative tumor diameter was 3.9 ± 1.27 cm, and 97 (78.2%) patients had BM size greater than 3 cm. A total of 50 (40.3%) patients were treated using TT. Targeted agents used were 15 for EGFR, 11 for HER2, 8 for ER/PR, 6 for VEGF, 4 for PD-1, 2 for ALK, 2 for mTOR, and 2 for PARP (Table 1).

### 3.2. Overall Survival (OS)

As of May 2021, 84 (67.7%) of these patients were dead. The median follow-up duration was 45.8 months (95% CI: 35.6–56.0) by reverse Kaplan–Meier estimate. The median OS of entire cohort was 21.1 months (95% CI: 15.8–26.4). The Kaplan–Meier estimate for survival probability was 69.4% at 12 months and 49.2% at 24 months.

In univariable analysis, higher (≥90) KPS score (HR 0.53, 95% CI: (0.27–1.03), *p* = 0.062), TT (HR 0.43, 95% CI: (0.25–0.75), *p* = 0.003), controlled systemic disease (HR 0.58, 95% CI: (0.34–0.98), *p* = 0.042), and single brain metastasis (HR 0.61, 95% CI: (0.36–1.04), *p* = 0.07) were associated with longer OS.

**Figure 1:**
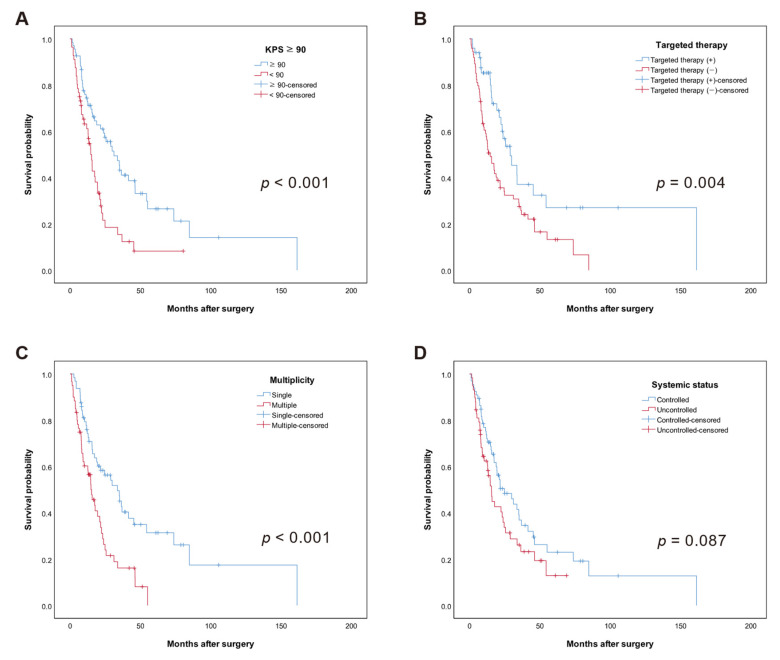
Kaplan–Meier curves of overall survival (OS) in months. The median OS in each group was as follows: (**A**) KPS score, median survival was 31.2 vs. 14.7, (KPS ≥ 90 vs. KPS < 90, *p* < 0.001), (**B**) use of targeted therapy, median OS was 28.9 vs. 13.4 (*p* = 0.004), (**C**) multiplicity of brain metastases, median OS was 33.7 vs. 14.9 (*p* < 0.001), (**D**) condition of systemic control, median OS was 24.9 vs. 15.6 (*p* = 0.006).

Kaplan–Meier curves of significant prognostic factors for OS are shown in Figure 1. Median OS was 31.2 months (95% CI: 21.2–41.2 months) in the KPS score ≥ 90 group but 14.7 months (95% CI: 11.9–17.5 months) in the KPS < 90 group. Median OS was 28.9 months in the TT (+) group (95% CI: 18.2–39.6 months) and 14.7 months (95% CI: 9.6–19.8 months) in the TT (–) group. Median OS was 33.7 months in the single BM group (95% CI: 22.1–45.3 months) and 14.9 months (95% CI: 10.2–19.5 months) in the multiple BM group. Median OS was 24.9 months in the systemic controlled group (95% CI: 12.8–36.9 months) and 15.6 months (95% CI: 12.4–18.8) in the systemic uncontrolled group (*p* = 0.087). There was no significant difference in OS by type or status of postoperative adjuvant radiation therapy (*p* = 0.923). With regard to primary site, the longest OS was observed for gynecologic cancer (46.2 months, 95% CI: 11.7–80.7), followed by breast cancer (24.6 months, 95% CI: 14.6–34.6), gastrointestinal (21.8 months, 95% CI: 0–51.2), lung (21.7 months, 95% CI: 16.0–27.5), head and neck (10.4 months), hepatobiliary (8.4 months, 95% CI: 0.9–15.8), and genitourinary (6.6 months, 95% CI: 4.0–9.2) (*p* = 0.289).

In multivariable analysis, higher (≥90) KPS score (HR 0.53, 95% CI: 0.32–0.87, *p* = 0.011), TT (HR 0.43, 95% CI: 0.26–0.70, *p* = 0.001), controlled systemic disease (HR 0.63, 95% CI: 0.40–1.00, *p* = 0.047), and single brain metastasis (HR 0.55, 95% CI: 0.34–0.90, *p* = 0.016) were associated with longer OS (Table 2).

### 3.3. Local Control (LC)

**Figure 2:**
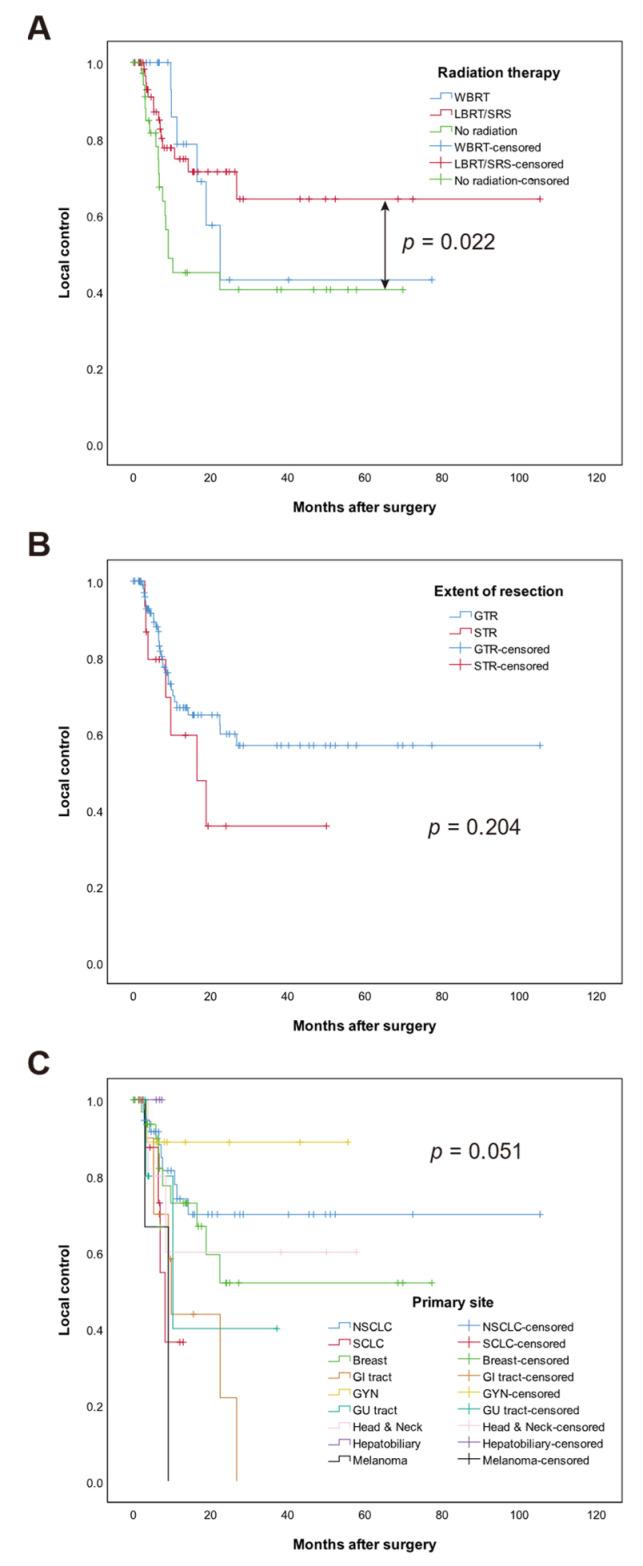
Kaplan–Meier curves of local control (LC) in months among 124 patients. The median LC values in each group were as follows: (**A**) radiation therapy, mean local recurrence-free survival time was 71.6 vs. 40.4 vs. 33.7, (LBRT/SRS vs. WBRT vs. No radiation, respectively *p* = 0.022) (**B**) extent of resection, mean local recurrence-free survival time was 64.4 vs. 24.6 (GTR vs. STR), respectively (*p* = 0.204), (**C**) primary site of brain metastases (BM) (*p* = 0.051).

During the analysis period, 37 (29.8.%) patients experienced postoperative local recurrence at the resection cavity. LC probability was 75.0% at 12 months and 71.0% at 24 months in the entire cohort. Gross total resection was associated with higher LC in univariable (HR 0.29, 95% CI: 0.11–0.83, *p* = 0.021) and multivariable analyses (HR 0.29, 95% CI: 0.11–0.78, *p* = 0.014) (Table 3). Origin of cancer (*p* = 0.041) was associated with LC in multivariable analysis. Both WBRT and LBRT/SRS showed superior LC than no radiation (HR 0.32, 95% CI 0.11–0.92, *p* = 0.034 and HR 0.38, 95% CI 0.17–0.85, *p* = 0.018, respectively). There was no significant difference in LC between WBRT and LBRT/SRS (*p* = 0.768). The 12-month LC was 56.8% in the no radiation group, 81.9% in the LBRT/SRS group, and 83.3% in the WBRT group. Kaplan–Meier curves of significant factors for LC are shown in Figure 2.

### 3.4. Distant Control (DC)

**Figure 3:**
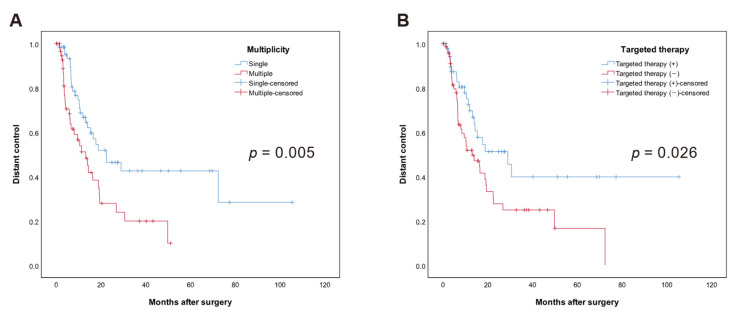
Kaplan–Meier curves of distant control (DC) in months among 124 patients. (**A**) Median distant-free survival time was 22.4 vs. 13.2 for single vs. multiple brain metastases, respectively (*p* = 0.005), (**B**) Median distant-free survival time was 29.0 vs. 13.0 with use of targeted therapy vs. no use of targeted therapy (*p* = 0.026).

A total of 61 patients (49.2%) had developed new BM outside of the resected region during the follow-up period. The 12 months distant brain control was 66.9%; 24 months was 54.8%. Median DC was 16.5 months (95% CI: 11.9–21.2) for the entire cohort. In univariate analysis, using TT (HR 0.45, 95% CI: 0.23–0.88, *p* = 0.02) and single brain metastasis (HR 0.53, 95% CI: 0.28–0.98, *p* = 0.044) were associated with higher DC (Table 4). In multivariate analysis, using TT (HR 0.54, 95% CI: 0.32–0.91, *p* = 0.022) and single brain metastasis (HR 0.47, 95% CI: 0.29–0.79, *p* = 0.004) were associated with higher DC. Kaplan–Meier curves of significant factors for DC are shown in Figure 3.

### 3.5. Leptomeningeal Metastases (LMM)

In total, 27 patients (21.8%) developed radiologically or cytologically confirmed LMM at a median of 72.4 months (95% CI: 2.2–142.6 months). Melanoma and breast cancer had the highest incidence of LMM (66.7% and 38.2%, respectively), but the difference was not significant (*p* = 0.293). There was no significant factor identified in the univariable analysis. However, multivariable analysis revealed that synchronous BM (HR 0.25, 95% CI: 0.08–0.86, *p* = 0.028), TT (HR 0.42, 95% CI: 0.18–0.95, *p* = 0.038), and controlled systemic disease (HR 0.44, 95% CI: 0.20–0.99, *p* = 0.047) were associated with lower incidence of LMM (Table 5).

## 4. Discussion

There have been many studies on postoperative survival and recurrence of BM, but not many have included TT as a prognostic factor. Our study was the first to analyze survival and recurrence after resection of BM with both TT and WBRT. The results suggest that the use of TT is associated with longer survival, better DC, and a lower risk of LMM.

In the updated Graded Prognostic Assessment (GPA), which is the most reliable prognostic index for brain metastases, molecular markers with available TT were significant prognostic factors in breast, non-small cell lung cancer (NSCLC), and melanoma [21,22,23]. Our data suggest that TT remains prognostic in the subset of patients who underwent surgical treatment for BM.

It is well known that a higher KPS score is related to a better prognosis of BM patients [24,25]. KPS score is the most important prognostic factor used to calculate all known prognostic scoring systems for BM, including GPA, recursive partitioning analysis (RPA), the score index for radiosurgery (SIR), and the basic score for brain metastases (BSBM) [21,26,27,28,29]. In this study, it was confirmed that the OS was a significant factor in the patient group with KPS score ≥ 90 (*p* = 0.011 in multivariate). Additionally, prognostic factors for OS were status of systemic disease and the number of metastatic brain tumors, as previously reported in other studies [23,30]. Single metastasis was significantly associated with better OS (*p* = 0.016).

Prognostic factors affecting postoperative local recurrence in patients with metastatic brain tumors are known to be extent of resection and postoperative WBRT or SRS [30,31]. In our study, both WBRT (*p* = 0.034) and LBRT/SRS (*p* = 0.018) were associated with better LC than was no radiation. Gross total resection also showed better LC (*p* = 0.014). The effect of TT on LC was not significant (*p* = 0.335), possibly due to a small number (29.8%) of patients who had local recurrence during the follow-up period.

Previous studies have shown that single BM and WBRT were favoring factors for DC after surgery in patients with BM [31]. In our study, TT (*p* = 0.022) and single metastatic cancer (*p* = 0.004) were significant for high DC. WBRT did not seem to compensate for the prognostic disadvantage of multiple metastases in our study. No other studies have revealed the effect of TT on DC after surgery of metastatic brain tumors. Given that TT is a treatment that inhibits the proliferation of tumor cells by interfering with specific target molecules, it is plausible that TT can improve DC.

In our study, LC and DC were 75.0% and 66.9% at 12 months, respectively. In particular, 26.3 months after the surgery, there was no additional local recurrence. These results indicate that BM itself can be well controlled with appropriate treatment, and that it is due to systemic disease progression that affects OS. This is consistent with the findings of this study, where the presence of systemic control (*p* = 0.027) is significant to OS.

Patients with BM who need surgery typically have a poor prognosis [32]. However, since the introduction of TT, more patients survive for a long time even if they have BM, and the follow-up period has been prolonged [33]. TT also has been shown to affect BM [34,35,36]. Newer-generation tyrosine kinase inhibitors are thought to be more effective against BMs because they are more permeable to the blood–brain barrier [37]. Based on this assumption, clinical trials of BM patients with TT are ongoing. The OUTRUN trial is a randomized phase II trial that compares TT only and TT plus SRS (ClinicalTrials.gov identifier: NCT03497767). The ORBITAL trial includes BM patients even if they have LMM (ClinicalTrials.gov identifier: NCT04233021).

The OS of patients with BM who have undergone surgery was not related to the type of radiation treatments, including SRS and WBRT [38]. Our study showed that LBRT/SRS has advantages in terms of LC over no radiation. Previously, when the survival was short after the onset of BM in a cancer patient, decline in cognitive function caused by WBRT was rarely noticed. However, as cancer treatment developed and prolonged survival, local treatments such as SRS rather than WBRT were used to improve the quality of life [39], which had no adverse effect on cognition but an equivalent LC effect as WBRT [40].

Postoperative WBRT reduces the possibility of LMM [41]. However, no survival benefit was proven in previous studies with postoperative WBRT [6]. Our study also did not show a significant difference in the incidence of LMM between LBRT/SRS and WBRT groups (*p* = 0.493). Interestingly, use of TT was related to a lower incidence of LMM (*p* = 0.038) in our study. To the best of our knowledge, this finding is original. This result suggests that TT can suppress the development of LMM. The other possibility is that the intrinsic nature of cancer differs among subtypes, with some having greater possibility to seed LMM [42]. Another explanation for this finding would be “just chance” due to small patient numbers, which is the one of limitations of this study. Nonetheless, TT had a survival benefit for patients with LMM with EGFR-mutated NSCLC [43,44]. For wild-type EGFR NSCLC, WBRT performed even after confirmation of LMM survival benefit in a retrospective study [41]. Taken together, these findings suggest that WBRT can be deferred until LMM occurs.

SRS in the resection cavity can be administered either as a single session or in fractions. However, it has not been revealed which of the two methods is more effective in LC for BM. For large unresected BM, fractionated SRS is known to have better LC and lower radiation necrosis than single session SRS [45]. Similar to in cavity radiosurgery, fractionation enables larger target volume with the same BED enabling a 2 mm margin radiation, which is known to improve LC [46,47]. Thus, fractionated SRS is expected to play a major role in postoperative SRS.

This study is limited in that it presented an analysis of the results in a relatively small number of patients with limited distribution as a retrospective study in a single institution. Selection bias cannot be ruled out. We do not know which specific TT had the greatest effect on survival. Given that 34 patients received TT for EGFR, HER2, or ER/PR and 16 patients received other TT, the better OS reported for TT is possibly due to effects seen in the EGFR/HER2/ER/PR group. However, because the number of patients using each TT was small, the differences in effectiveness between the TTs were not significant. Further prospective studies in a large-scale cohort are mandatory to reveal the efficacy of TT and localized radiotherapy on the survival and quality of life in patients with BM.

## 5. Conclusions

This analysis suggests that TT improved the survival of patients who had undergone surgical resection of BM. We reaffirmed that well-known prognostic factors such as KPS, controlled primary cancer, and number of BMs are significant for survival in this cohort. We did not observe any differences in OS, DC, or LMM for WBRT versus LBRT/SRS. However, this result should be interpreted with caution due to the limited number of cases. Given that longer survival is achieved with systemic therapy rather than radiation, more lesion-targeted radiotherapies that avoid neurotoxicity are reasonable choices. Targeting both molecular and stereotactic targets will be key to maximizing the survival of BM patients while maintaining their quality of life.

## Figures and Tables

**Table 1:** Patient characteristics.

Characteristic	Count (%)
Age	59 (28–78) *
Sex	
Men	59 (47.6)
Women	65 (52.4)
Postoperative radiotherapy	
LBRT/SRS	63 (50.8)
WBRT	24 (19.4)
No radiation	37 (29.8)
Karnofsky Performance Scale	90 (30–100) *
≥90	68 (54.8)
<90	56 (45.2)
Primary site	
Non-small cell lung cancer	41 (33.1)
Small cell lung cancer	9 (7.3)
Breast	34 (27.4)
Gastrointestinal	12 (9.7)
Gynecologic	10 (8.1)
Genitourinary	6 (4.8)
Head and Neck	5 (4.0)
Hepatobiliary	4 (3.2)
Melanoma	3 (2.4)
Synchronicity	
Metachronous	91 (73.4)
Synchronous	33 (26.6)
Tumor size	3.75 cm (1.3–7.6 cm) *
≥3cm	97 (78.2)
<3cm	27 (21.8)
Targeted therapy	
Targeted therapy (+)	50 (40.3)
EGFR	15
HER2	11
ER/PR	8
VEGF	6
PD-1	4
ALK	2
mTOR	2
PARP	2
Targeted therapy (−)	74 (59.7)
Systemic control	
Controlled	66 (53.2)
Uncontrolled	58 (46.8)
Extent of resection	
Gross total resection	109 (87.9)
Subtotal resection	15 (12.1)
Multiplicity	
Single	64 (51.6)
Multiple	60 (48.4)

LBRT, local brain radiotherapy; SRS, stereotactic radiosurgery; WBRT, whole-brain radiotherapy; EGFR, epidermal growth factor receptor; HER2, human epidermal growth factor receptor 2; ER, estrogen receptor; PR, progesterone receptor; VEGF, vascular endothelial growth factor; PD-1, programmed cell death protein 1; ALK, Anaplastic lymphoma kinase; mTOR, mammalian target of rapamycin; PARP, Poly (ADP-ribose) polymerase; * Median (range).

**Table 2:** Univariable and multivariable Cox regression for overall survival.

Overall Survival	Univariable		Multivariable	
Parameters	HR (95% CI)	*p*	HR (95% CI)	*p*
Age (per 1 increase)	1.00 (0.97–1.02)	0.795		
Sex (Women: Men)	0.73 (0.32–1.68)	0.464		
Radiation method				
No radiation	1			
WBRT	1.15 (0.56–2.36)	0.701		
LBRT/SRS	1.07 (0.48–2.37)	0.864		
KPS ≥ 90	0.53 (0.27–1.03)	0.062	0.53 (0.32–0.87)	0.011
Primary site	-	0.701		
Synchronicity	0.85 (0.46–1.60)	0.621		
Size (≥3 cm: <3 cm)	1.11 (0.62–1.99)	0.737		
Targeted therapy	0.43 (0.25–0.75)	0.003	0.43 (0.26–0.70)	0.001
Systemic control	0.58 (0.34–0.98)	0.042	0.63 (0.40–1.00)	0.047
Gross total resection	1.00 (0.48–2.07)	0.996		
Single metastasis	0.61 (0.36–1.04)	0.070	0.55 (0.34–0.90)	0.016

LBRT, local brain radiotherapy; SRS, stereotactic radiosurgery; WBRT, whole-brain radiotherapy; KPS, Karnofsky performance scale.

**Table 3:** Univariable and multivariable Cox regression for local control.

Local Control	Univariable		Multivariable	
Parameters	HR (95% CI)	*p*	HR (95% CI)	*p*
Age (per 1 increase)	0.99 (0.95–1.03)	0.469		
Sex (Women: Men)	0.92 (0.28–3.00)	0.883		
Radiation method				
No radiation	1		1	
WBRT	0.21 (0.06–0.68)	0.009	0.32 (0.11–0.92)	0.034
LBRT/SRS	0.37 (0.15–0.88)	0.025		0.018
KPS ≥ 90	0.59 (0.22–1.63)	0.31		
Primary site				
Non-small cell lung cancer	1			
Small cell lung cancer	2.15 (0.56–8.28)	0.266	3.46 (1.02–11.72)	0.047
Breast	0.85 (0.20–3.68)	0.823	1.01 (0.39–2.64)	0.983
Gastrointestinal	3.30 (1.14–9.59)	0.028	3.83 (1.39–10.56)	0.009
Gynecologic	0.40 (0.04–4.04)	0.433	0.40 (0.05–3.14)	0.38
Genitourinary	0.45 (0.07–2.90)	0.398	1.12 (0.22–5.68)	0.892
Head and neck	0.33 (0.04–2.55)	0.287	0.34 (0.06–1.96)	0.227
Hepatobiliary	-	0.981	-	0.981
Melanoma	3.01 (0.52–17.43)	0.219	3.88 (0.78–19.37)	0.098
Synchronicity	0.47 (0.17–1.26)	0.133	0.44 (0.18–1.07)	0.071
Size (≥3 cm: <3 cm)	0.76 (0.32–1.81)	0.539		
Targeted therapy	0.64 (0.26–1.58)	0.335		
Systemic control	0.96 (0.44–2.09)	0.91		
Gross total resection	0.30 (0.11–0.83)	0.021	0.29 (0.11–0.78)	0.014
Single metastasis	0.95 (0.40–2.26)	0.905		

LBRT, local brain radiotherapy; SRS, stereotactic radiosurgery; WBRT, whole-brain radiotherapy; KPS, Karnofsky performance scale.

**Table 4:** Univariable and multivariable Cox regression for distant control.

Distant Control	Univariable		Multivariable	
Parameters	HR (95% CI)	*p*	HR (95% CI)	*p*
Age (per 1 increase)	0.98 (0.95–1.01)	0.184		
Sex (Women: Men)	0.68 (0.27–1.71)	0.411		
Radiation method				
No radiation	1			
WBRT	0.86 (0.34–2.18)	0.756		
LBRT/SRS	1.41 (0.69–2.89)	0.349		
KPS ≥ 90	0.65 (0.30–1.38)	0.261		
Primary site	-	0.865		
Synchronicity	0.80 (0.38–1.66)	0.542		
Size (≥3 cm; <3 cm)	1.35 (0.71–2.58)	0.355		
Targeted therapy	0.45 (0.23–0.88)	0.020	0.54 (0.32–0.91)	0.022
Systemic control	0.70 (0.37–1.32)	0.265		
Gross total resection	0.71 (0.34–1.52)	0.384		
Single metastasis	0.53 (0.28–0.98)	0.044	0.47 (0.29–0.79)	0.004

LBRT, local brain radiotherapy; SRS, stereotactic radiosurgery; WBRT, whole-brain radiotherapy; KPS, Karnofsky performance scale.

**Table 5:** Univariable and multivariable Cox regression for leptomeningeal metastases.

Leptomeningeal Metastases	Univariable		Multivariable	
Parameters	HR (95% CI)	*p*	HR (95% CI)	*p*
Age (per 1 increase)	1.03 (0.96–1.09)	0.430		
Sex (Women: Men)	3.98 (0.43–37.31)	0.226		
Radiation method				
No radiation	1			
WBRT	1.72 (0.36–8.13)	0.493		
LBRT/SRS	1.31 (0.41–4.15)	0.646		
KPS ≥ 90	1.23 (0.36–4.18)	0.741		
Primary site	-	0.293		
Synchronicity	0.29 (0.07–1.26)	0.099	0.25 (0.08–0.86)	0.028
Size (≥3 cm; <3 cm)	0.65 (0.21–2.02)	0.458		
Targeted therapy	0.46 (0.16–1.33)	0.153	0.42 (0.18–0.95)	0.038
Systemic control	0.55 (0.21–1.44)	0.223	0.44 (0.20–0.99)	0.047
Gross total resection	0.79 (0.24–2.66)	0.707		
Single metastasis	0.47 (0.19–1.17)	0.105	0.47 (0.22–1.02)	0.055

LBRT, local brain radiotherapy; SRS, stereotactic radiosurgery; WBRT, whole-brain radiotherapy; KPS, Karnofsky performance scale.

## Data Availability

No new data were created or analyzed in this study. Data sharing is not applicable to this article.
